# Sustained Cerebrovascular and Cognitive Benefits of Resveratrol in Postmenopausal Women

**DOI:** 10.3390/nu12030828

**Published:** 2020-03-20

**Authors:** Jay Jay Thaung Zaw, Peter R. C. Howe, Rachel H. X. Wong

**Affiliations:** 1 School of Biomedical Sciences and Pharmacy, University of Newcastle, Callaghan 2308, New South Wales, Australia; JayJay.ThaungZaw@uon.edu.au (J.J.T.Z.); peter.howe@newcastle.edu.au (P.R.C.H.); 2 Institute for Resilient Regions, Springfield Central, University of Southern Queensland, Springfield Central 4300, Queensland, Australia; 3 School of Health Sciences, University of South Australia, Adelaide 5000, South Australia, Australia

**Keywords:** resveratrol, ageing, menopause, cognitive decline, cerebrovascular function, neurovascular coupling, phytoestrogen, nutraceutical

## Abstract

Deficits in the cerebral microcirculation contribute to age-related cognitive decline. In a pilot study of postmenopausal women, we found that supplementation with a low dose of resveratrol, a phytoestrogen, for 14 weeks improved cerebrovascular and cognitive functions. We have since undertaken a larger, longer term study to confirm these benefits. Postmenopausal women aged 45–85 years (*n* = 129) were randomized to take placebo or 75 mg trans-resveratrol twice daily for 12 months. Effects on cognition, cerebral blood flow, cerebrovascular responsiveness (CVR) and cardiometabolic markers (blood pressure, diabetes markers and fasting lipids) were assessed. Compared to placebo, resveratrol improved overall cognitive performance (*P* < 0.001) and attenuated the decline in CVR to cognitive stimuli (*P* = 0.038). The latter effect was associated with reduction of fasting blood glucose (r = −0.339, *P* = 0.023). This long-term study confirms that regular consumption of resveratrol can enhance cognitive and cerebrovascular functions in postmenopausal women, with the potential to slow cognitive decline due to ageing and menopause.

## 1. Background

Since 1990, the number of people globally living with dementia has more than doubled. This is mainly due to increased population growth and ageing [1]. Importantly, independent of life expectancy, dementia mortality rates in women in 2016 were almost twice that of men. This may be partly attributable to the abrupt decline of estrogen at menopause and the associated loss of its protective effects on cardiovascular [2] and neural functions [3]. 

Estrogen activates estrogen α and β receptors (ER) on endothelial cells to facilitate vasodilatation by increasing endothelial nitric oxide (NO). Thus, estrogen deprivation can accelerate age-related arterial stiffening and impair tissue perfusion by reducing endothelium-dependent vasodilatation. This not only increases the risk of cardiovascular disease postmenopausally [4] but also reduces cerebrovascular responsiveness (CVR) in postmenopausal women compared to pre-menopausal women and men [5,6]. Reduced cerebral blood flow (CBF) and CVR are associated with cognitive impairment [7]. We have also reported that reduced CVR during mental task activation (neurovascular coupling) is predictive of poor cognitive performance in postmenopausal women [8]. A meta-analysis has shown that postmenopausal women perform worse on verbal memory and executive function tests compared to peri-menopausal women [9]. Therefore, maintaining the health of the cerebral vasculature may slow cognitive decline in postmenopausal women. 

Resveratrol (3,5,4′-trihydroxy-trans-stilbene), a phytoestrogen present in a variety of foods such as grapes, berries and nuts, has been shown to improve endothelial vasodilator function in humans [10]. Resveratrol can act through multiple mechanisms including activation of endothelial ER to increase NO production and thereby facilitate endothelium-dependent vasodilatation necessary for adequate cerebral perfusion [11]. Resveratrol has been shown to improve verbal memory in older adults after six months of 200 mg of supplementation per day compared to placebo [12]. Using functional magnetic resonance imaging, Witte et al. found that resting-state functional connectivity in the hippocampus (a region critical for memory functions) and other brain regions was significantly increased in the resveratrol group and was correlated with improvement in verbal memory [12]. Kennedy et al. reported that compared to placebo, acute doses of 250 mg and 500 mg of resveratrol resulted in dose-dependent increases of resting CBF in younger adults, although cognitive function was not affected [13]. In a dose-response evaluation, Wong et al. found that 75 mg resveratrol, the lowest dose tested, was the most efficacious dose to acutely elicit global cerebral vasodilatation [14] and improve performance of a sustained attention task in type 2 diabetes patients [15]. In a subsequent pilot study of chronic resveratrol treatment (2 × 75 mg/day for 14-weeks) in 80 postmenopausal women, we observed an improvement of cognitive performance, which was accompanied by enhanced neurovascular coupling, suggesting that regular resveratrol supplementation might attenuate accelerated cognitive ageing in a vulnerable population by helping to maintain normal circulatory function [16]. We now intend to investigate whether these benefits can be sustained long-term as well as examining its effects on a wide range of cardiometabolic markers. 

## 2. Subjects and Methods

### 2.1. Study Design

A 24-month randomised, double-blind, placebo-controlled (crossover comparison) dietary intervention trial was conducted to evaluate the effects of resveratrol supplementation (75 mg twice daily) on cognitive performance to a neuropsychological test battery and on associated cerebrovascular and cardiometabolic markers in post-menopausal women. This manuscript will report an interim analysis performed at the end of first stage of crossover to determine the within-individual treatment changes (post-pre supplementation) between the placebo and resveratrol arm. For 90% power to detect a statistically significant (*P* < 0.05) medium effect size (Cohen’s *d* = 0.5) improvement in the primary outcome (overall cognitive performance), 87 completers were required for a crossover comparison. To allow for 45% attrition due to a long-term study and to account for the difficulty in detecting an acoustic temporal window for cerebrovascular function assessments, especially in elderly women, we aimed to recruit 170 women. The trial was conducted at the Clinical Nutrition Research Centre of the University of Newcastle in New South Wales, Australia in accordance with the Declaration of Helsinki and the Principles of Good Clinical Practice as outlined by the International Conference on Harmonisation. The protocol was approved by the University of Newcastle’s Human Research Ethics Committee (H-2016-0091) and registered with the Australian and New Zealand Clinical Trial Registry (ACTRN12616000679482p). 

### 2.2. Study Population

From November 2016 to May 2017, community-dwelling postmenopausal women residing in the Hunter region of New South Wales were recruited through approved newspaper and radio campaigns and from a database of previous Clinical Nutrition Research Centre participants and from the Hunter Medical Research Institute Volunteer Registry. Interested volunteers were provided with a detailed information sheet and completed a health and lifestyle questionnaire to determine whether they met the inclusion criteria of the study. Eligible participants were aged 45–85 years, >12 months post-menopausal and willing to maintain their current lifestyle throughout the study. We did not include volunteers if they took hormone replacement therapy, insulin or warfarin within the past six months, had suspected dementia or clinical depression. To avoid possible drug interactions, hormonal influences and other pathological brain lesions causing cognitive impairments, individuals who had a history of breast or cervical cancer, major cardiovascular, kidney or liver disease or a neurological disorder were also excluded. 

### 2.3. Screening Visit and Follow-Up Assessments

Potentially eligible participants attended the screening/baseline visit with at least two hours fasting (no medication, food or caffeinated or sugary beverages). Written informed consent was obtained prior to any assessments. A clinical investigator measured height (m), weight (kg), and waist circumference at the narrowest part of midriff (cm). Body mass index (BMI) was derived by dividing weight by height^2^. Seated blood pressure (BP) was assessed after 10 minutes of rest and those above 160/100 mmHg were excluded. Participants’ global cognitive status was assessed using the Australian Version of Addenbrooke’s Cognitive Examination III (ACE-III), where the cut-off for suspected dementia is less than 82% [17]. Those who met full eligibility criteria were enrolled and invited for follow-up assessments. Participants were instructed to refrain from consuming their supplement on the day of any visit to assess the sustained effects of resveratrol. 

### 2.4. Investigational Product and Allocation

Resveratrol (Veri-te™) and placebo capsules were identical in shape and colour and supplied by Evolva SA, Switzerland. Each resveratrol capsule contained 75 mg of >98% pure synthetic trans-resveratrol and placebo comprised of several inert excipients. Containers of capsules were only identifiable by code numbers; an independent investigator who held the code allocated volunteers to resveratrol or placebo capsules using Altman’s randomization by minimisation procedure [18] in order to balance treatment groups based on age, postmenopausal years and clinic blood pressure (measured at the screening visit).

### 2.5. Outcome Assessments

#### 2.5.1. Clinic Blood Pressure and Arterial Compliance

Seated BP was taken with an appropriately sized cuff placed over the brachial artery of the non-dominant hand (Cardiovascular Profiler CR 2000, Minnesota, MN, USA). The dominant hand was stabilized by a rigid wrist support and a tonometer was attached perpendicularly over the radial artery to measure the arterial compliance (AC) of systemic arteries. Three repeated measures of BP and AC were performed at two-minute intervals. The first BP measurement was discarded and the remaining readings were averaged. 

#### 2.5.2. Cerebrovascular Function Assessments with Transcranial Doppler Ultrasound

##### Basal Cerebral Haemodynamics

A transcranial Doppler ultrasound (TCD) headpiece (Doppler-Box X, Singen, Germany) was fitted on participants’ heads with probes on the left and right temporal area to insonate middle cerebral arteries (MCA) at a depth of 45-60 mm. TCD is a non-invasive technique to assess the changes in blood flow velocities (BFV) in the brain [19]. A 30-second continuous recording of basal BFV (maximum, minimum, mean) was obtained before hypercapnic provocation and before the start of each cognitive test. Stiffness in the cerebral vessels or pulsatility index (PI) at baseline was derived as follows: (maximum BFV − minimum BFV)/mean BFV. 

##### Cerebrovascular Responsiveness (CVR)

CVR to hypercapnia: Participants breathed in carbogen gas (95% O_2_, 5% CO_2_) through a two-way non-rebreathing mouthpiece for 180 seconds. TCD recorded the increases in bilateral beat-to-beat mean BFV throughout the hypercapnic challenge. A further 60 seconds recording was taken whilst the participants inhaled normal room air to ensure that their BFV returned to resting values. 

CVR to cognitive stimuli (neurovascular coupling): The TCD was kept in position throughout the neuropsychological test battery to assess CVR to cognitive stimuli, and it was recorded before (30-second baseline) and during each cognitive task. 

CVR recordings were smoothed and analysed in TableCurve^TM^ (TableCurve 2D by Systat Software Inc., San Jose, CA, USA) using data spline estimation with Loess at 20% for hypercapnia assessments and 10% for neurovascular coupling to determine the peak increase in mean BFV. CVR was calculated as follows: [(peak mean BFV − resting mean BFV)/resting mean BFV × 100].

#### 2.5.3. Cognitive Performance

The cognitive test battery consisted of seven cognitive tests from the National Institutes of Health Toolbox (NIH-ToolBox) assessment [20] and three other validated cognitive tests viz. Rey’s Auditory Verbal Learning Test (RAVLT), Forward Spatial Span Test and Trail Making Task. Relevant tests chosen for cognitive domains were depicted in Table 1. This battery provided different versions of the tests to administer at baseline and after 12 months, thus avoiding practice effects. 

A trained study investigator delivered the test battery (except for the Trail Making Task) on an iPad, which took about 90 minutes to complete. Data were stored in the device, transferred securely and backed-up every week. Practice runs preceded each test to ensure the participants’ full understanding of the instructions. 

#### 2.5.4. Blood Biomarker Assessment

Overnight fasted venous blood samples were collected by a phlebotomist at baseline and after 12 months. The biomarker analysis included fasting serum glucose, insulin, lipids (total cholesterol, triglycerides, HDL and LDL-cholesterol) and high-sensitivity C-reactive protein (hs-CRP) as a marker of systemic inflammation. Homeostatic Model Assessment of Insulin Resistance (HOMA-IR) was derived from the fasting glucose and insulin results.

### 2.6. Intervention

Participants were instructed to take two capsules of their allocated treatment each day (one in the morning and one in the evening). A supplement diary was provided to record the time that the capsules were taken and any changes to medications or habitual lifestyle. If a dose was missed, participants were allowed to catch up on the same day but not to double-up the next day. There was a face-to-face compliance check at month-6 where the participants were required to attend an hour-long clinic visit to undergo BP and CVR assessments and acquire new supplement bottles and diaries. All unused capsules were returned at the six- and 12-month visits and were counted and tallied with supplement diaries to monitor compliance. In addition, a study investigator made phone calls every three months to track participants’ well-being, lifestyle or medication changes and any occurrence of illness or side effects.

### 2.7. Statistical Analysis

Treatments by time effects were determined by analysis of variance (ANOVA) using SPSS version 25.0 (SPSS by IBM Inc. Chicago, IL, USA). The primary outcome was the treatment change from baseline in cognitive performance, determined using a composite score (z-scores) of the neuropsychological test battery between resveratrol and placebo groups. The scores from each cognitive task obtained at 12 months were converted to z-scores derived from the cohort’s performance at baseline. Effect sizes were calculated using Cohen’s *d* [21]. Secondary outcomes were the treatment by time effects on BFV, PI, CVR to hypercapnia, CVR to cognitive stimuli and cardiometabolic markers. Pearson’s correlational analysis was applied to examine the associations between cerebrovascular function and other outcome measures. The Benjamini–Hochberg procedure [22] was used to correct P-values for secondary outcomes (cerebrovascular function and cardiometabolic markers) to minimise type I errors; the false discovery rate was set at 0.15. Data are presented as mean ± SEM (standard error of the mean) unless otherwise stated.

## 3. Results

### 3.1. Participant Disposition

**Figure 1:**
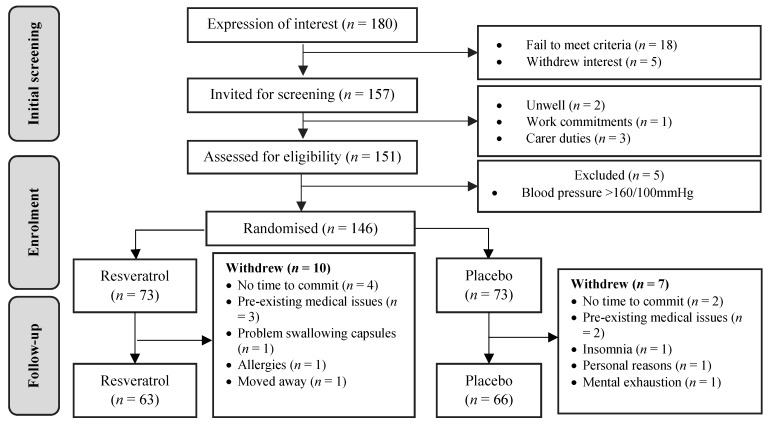
CONSORT diagram. Flow of participants from initial contact until final assessment.

Figure 1 depicts the participant disposition of this study in accordance with CONSORT (Consolidated Standards of Reporting Trials) statement 2010 [23]. Although we formerly aimed to recruit 170 participants, due to logistical limitations, we invited 151 women for a screening visit and enrolled 146 participants, of which 129 participants completed the 12-month trial and were included in the analysis of the primary outcome. This provided 80% power at alpha 0.05, to detect a statistically significant medium effective size improvement (Cohen’s *d* = 0.5) in the primary outcome for a parallel comparison. Of 17 participants (12%) who withdrew their participation before 12 months, 10 had been allocated resveratrol and seven placebo. 

### 3.2. Baseline Characteristics

Table 2 shows baseline characteristics of 146 women who were enrolled and randomized to placebo (*n* = 73) and resveratrol (*n* = 73) groups. Averaging 64 years of age and 15 years post-menopausal, they were slightly overweight, normotensive, well-educated and cognitively unimpaired, as indicated by ACE-III scores. There were no differences between groups. 

### 3.3. Systemic Vascular Function

Blood pressure and systemic arterial compliance (elasticity of both large and small arteries) remained unchanged after 12 months supplementation in both treatment groups (see Supplementary Materials Table S1). 

### 3.4. Cognitive Performance

**Figure 2:**
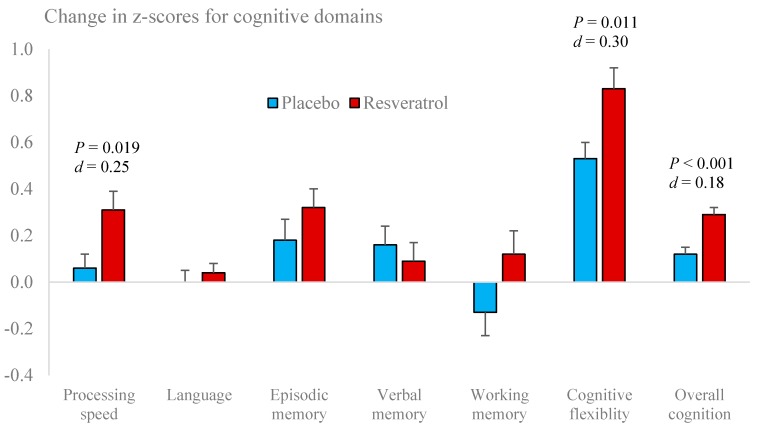
Performance changes in cognitive domains following placebo and resveratrol treatments.

Performance of the Pattern Comparison Speed test improved with resveratrol supplementation compared to placebo. There were no other significant changes in individual tests (Table 3). However, improvements were seen in two cognitive domains, viz. processing speed (*P* = 0.019, Cohen’s *d* = 0.25) and cognitive flexibility (*P* = 0.011, Cohen’s *d* = 0.30), resulting in a modest increase in overall cognitive performance (*P* = < 0.001, Cohen’s *d*= 0.18) (Figure 2). 

Improvements of overall cognitive performance in the resveratrol-treated group were greater in those individuals with lower cognitive performance at baseline (R = −0.380, *P* = 0.002).

### 3.5. Cerebrovascular Function

**Figure 3:**
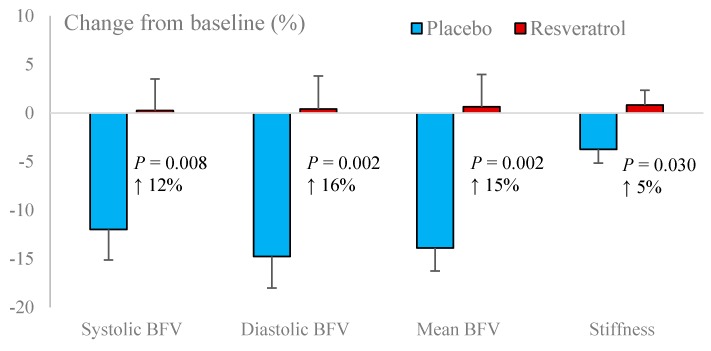
Changes in basal blood flow velocity (BFV) and pulsatility index (arterial stiffness) following resveratrol and placebo treatments.

**Figure 4:**
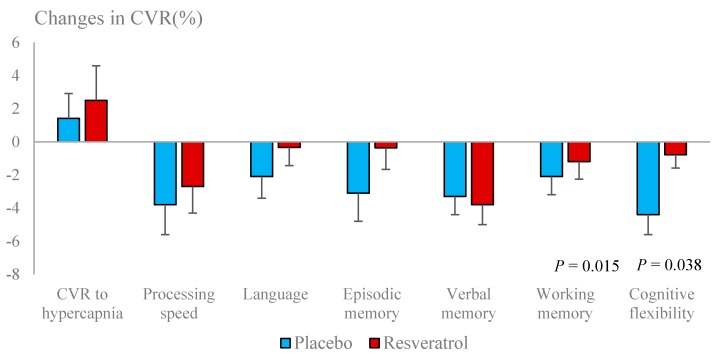
Changes in cerebrovascular responsiveness (CVR) to hypercapnia and to cognitive tests following resveratrol and placebo treatments.

Basal BFV (systolic, mean and diastolic) and PI were significantly improved with resveratrol (Figure 3). CVR to hypercapnia was unaffected by resveratrol but a decline in neurovascular coupling capacity was attenuated by resveratrol (Table 4). Overall neurovascular coupling capacity, especially in response to tests of cognitive flexibility, was improved (Figure 4). 

### 3.6. Cardiometabolic Markers

**Figure 5:**
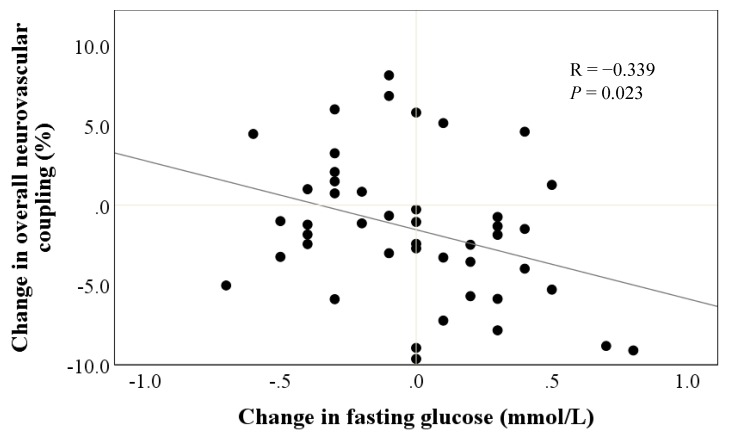
Association between treatment change in fasting glucose and treatment change in overall neurovascular coupling following resveratrol supplementation.

There were no significant differences in cardiometabolic markers between placebo and resveratrol groups (Table 5). However, we observed that reductions in fasting blood glucose were associated with the improvements in overall neurovascular coupling capacity following resveratrol supplementation (r = −0.339, *P* = 0.023) (Figure 5). 

### 3.7. Adverse Events

A total of 12 adverse events were reported during the trial, four of which were serious conditions requiring hospitalisation, viz. a urinary tract infection, a bowel blockage, an oesophageal tear repair and a pre-scheduled operation on the lumbar spine. All four occurred in the placebo group. Of the remaining eight adverse events, four occurred in the resveratrol group but were not necessarily attributable to supplementation (viz. itching, menses, prolapsed bladder and a pre-scheduled left eye operation) and four occurred in the placebo group (viz. itching, exacerbation of gastric reflux, constipation and a pre-scheduled breast reduction procedure). Treatment compliance averaged 95% in both groups. 

## 4. Discussion

In this 12-month trial, we sought to confirm the unique findings of our 14 week pilot study in postmenopausal women [16] and ascertain whether the benefits of low-dose resveratrol on cerebrovascular and cognitive functions could be sustained with long-term supplementation.

We observed an improvement in overall cognitive performance which appeared to be due to improvements in processing speed and cognitive flexibility. Both processing speed and cognitive flexibility are part of executive function that requires speed, perceptual reasoning and accuracy to accomplish tasks [24]. After peaking in the third decade of life, executive function declines at an estimated annual rate of -0.02 standard deviations [25]. In fact, slowing of processing speed and mental flexibility are among the first cognitive changes reported in healthy older adults [24]. This “slowing” can negatively affect performance on other neuropsychological tests such as verbal fluency [24]. Our observed improvement in overall cognitive performance with resveratrol (*d* = 0.18) could potentially reverse cognitive ageing by up to 10 years. Therefore, optimising executive function in healthy older adults may delay subsequent impairment across other cognitive domains. 

Apart from our pilot study [16], there are only two clinical trials on cognitive effects of chronic resveratrol supplementation [12,26]. A six-month study by Witte et al. showed improvement of verbal memory in healthy older adults with a similarly low dose (200 mg/day) [12]. On the other hand, a study in young adults [26] reported a lack of interpretable cognitive effects following resveratrol supplementation (500 mg/day for a month), despite showing improvement of cerebrovascular function. This may be due to the high-performing cognitive status of this cohort, the shorter study duration or the higher dose of resveratrol given, bearing in mind that we have previously reported lower neurovascular coupling efficacy of resveratrol at higher doses [14]. 

We hypothesized that the observed cognitive benefits of resveratrol might be partly mediated by sustained improvement of endothelium-dependent vasodilator function, which modulates CBF during times of demand [11]. Supporting this, we found improved resting BFV, PI and attenuation of decline in neurovascular coupling following resveratrol supplementation. Allowing for normal ageing processes, cognitive decline and ultimately, dementia are linked to accelerated decline in resting CBF and CVR due to a decreased delivery of oxygen and nutrients in vulnerable brain regions such as the hippocampus [27]. In fact, a large population-based study in Rotterdam reported that healthy older adults with lower CBF measured by MRI performed significantly worse on tasks of information processing speed, executive and global cognitive function compared to those with higher CBF [28]. In addition, cerebral artery stiffness, marked by increased PI, is also associated with cognitive impairment [29] and predicts the progression from mild cognitive impairment to dementia [30]. Combination of low BFV and high PI can result in chronic hypoperfusion, which may cause progressive loss of neuronal function [31]. Given this evidence, our observation of improvements in resting BFV and PI with resveratrol highlights the ability of regular resveratrol supplementation to sustain cerebrovascular function, which may, in turn, preserve cognitive function in elderly women. 

Evidence has shown that resveratrol can modulate CBF through multiple mechanisms including activation of Sirtuin-1, adenosine-monophosphate protein kinase and ER α and β to increase endothelial NO synthase activity. This, in turn, increases NO production and bioavailability to facilitate vasodilator responsiveness and arterial smooth muscle relaxation during demand [11]. To assess the cerebral vasodilator response, hypercapnia provocation is commonly used to increase of blood CO_2_ concentration. The increase in BFV from resting values reflects dilation in the downstream microvasculature and thus is a good measure of global cerebral vasodilatation, independent of specific neuronal activation [32]. We did not observe enhancement of CVR to hypercapnia with resveratrol in this study, perhaps because cerebral vasodilator capacity was still optimal in our cohort of healthy elderly women. Older adults with established cardiovascular risk factors such as hypertension, dyslipidaemia or diabetes had lower CVR to hypercapnia compared to healthy older and young adults [32]. Moreover, impaired CVR to hypercapnia has been observed in individuals with mild cognitive impairment and Alzheimer’s disease [33], implicating cerebral hypoperfusion in the pathogenesis of cognitive impairment. 

Although we did not see any enhancement of CVR to hypercapnia, we found that resveratrol attenuated the decline of neurovascular coupling. Neurovascular coupling differs from CVR to hypercapnia as it represents localised changes in cerebral BFV in response to specific neuronal events. When neurons fire, the endothelium is activated to release NO, resulting in dilatation of local arterioles, which is detected as increased blood flow in the arteries supplying the activated brain region. Deficits in this synchronised action can lead to inadequate perfusion of critical brain regions, resulting in poor performance of cognitive tasks [34]. Therefore, impaired neurovascular coupling may be considered the beginning of a chain of events leading to a progressive decline in brain metabolism and cognition that characterizes dementia [35]. Identification of early cerebrovascular dysfunction has important diagnostic implications for future cognitive decline [36]. We have previously shown that poor cerebrovascular function is predictive of cognitive decline in healthy postmenopausal women [8]. 

We have previously shown that the beneficial effects of resveratrol on systemic vascular function are greater in those individuals with poorer vascular function at baseline [37]. This may account for the greater magnitude of improvement in neurovascular coupling seen in our pilot study of resveratrol supplementation in postmenopausal women [16] (d = 0.71), which elicited a greater improvement in overall cognitive performance (d = 0.69) than observed in the present study, as the participants in the pilot study had much lower overall neurovascular coupling at baseline. In this study, we have once again found that lower cognitive function at baseline is associated with greater improvements in overall cognitive performance by resveratrol. Taken together, regular resveratrol supplementation may be more beneficial for improving cognitive function in older adults with higher level of baseline endothelial dysfunction or cognitive impairments. The present study not only confirms the significant benefits of resveratrol seen in the pilot study but, most importantly, it shows that these benefits are not short-lived but can be sustained with ongoing supplementation for at least 12 months. Indeed, data from the placebo-treated group revealed a further decline of neurovascular coupling over 12 months, even in healthy older women. The present study demonstrates the potential of resveratrol to attenuate this decline, thereby protecting higher brain function in the elderly. 

We did not find any significant difference in systemic vascular function (BP, AC) or fasting glucose, insulin and lipids between the two treatments, although Timmers et al. had shown that resveratrol (150 mg/day for 30 days) can reduce plasma triglycerides and improve insulin sensitivity in obese male subjects (mean age: 52 ± 2 years, BMI: 31.6 ± 0.7 kg/m^2^) with low-grade chronic inflammation [38]. They also reported reduced inflammation; however, we did not observe an effect of resveratrol on hs-CRP, indicating that resveratrol is unlikely to alter cardiometabolic markers in elderly participants who are otherwise healthy (BMI: 25.6 ± 0.3 kg/m^2^) and without overt inflammation or other metabolic disturbances. 

Interestingly, we found a significant inverse relationship between the supplementation-induced changes in fasting glucose and overall neurovascular coupling. Our finding is consistent with Witte et al., who observed a reduction in HbA1C, the long-term biomarker of glucose control, which correlated with increased functional connectivity in the hippocampus assessed by functional MRI [12]. This is consistent with our previously published hypothesis that resveratrol might improve glucose uptake and/or reduce insulin demand by enhancing vasodilator function in skeletal muscle [39]. A recent meta-analysis has also shown that resveratrol can improve insulin sensitivity and insulin secretion in pancreatic β-cells and increase glucose uptake via Sirtuin-1 or adenosine monophosphate protein kinase mediated pathways [40]. Resveratrol could also facilitate expression of glucose transporter type-4 by acting on estrogen receptors on the endothelium and stimulate skeletal muscle glucose uptake [41], which could be an added metabolic benefit for postmenopausal women. Although we cannot rule out the exact mechanism of action, this finding could imply that resveratrol may induce improvements in energy metabolism through improvements of microcirculatory function, which could, in turn, protect neuronal function and counteract cognitive decline. 

This is the first long-term study of effects of resveratrol supplementation in postmenopausal women. The 12-month duration not only increases confidence in the sustainability of any benefits but it also eliminates any influences of seasonality on the outcomes. Whilst generating further evidence on the benefits of resveratrol for healthy ageing, there are some obvious limitations to its application. Individuals with higher health consciousness and cognitive awareness tend to volunteer for such intervention trials, which may influence their compliance and performance and ultimately, the interpretation of the results. Moreover, as our sample included healthy postmenopausal women only, the results cannot be generalized to the entire population. Further trials are necessary to determine the extent of potential benefit for men and for specific risk groups, e.g., hypertensives and diabetics, of both sexes.

## 5. Conclusions

Findings from this study confirm the results of our pilot study and demonstrate a sustained benefit of long-term low dose resveratrol supplementation, viz. the improvement of overall cognitive performance, which can be attributed, at least partly, to improvement of cerebral blood flow and vasodilator responsiveness during cognitive demands. We also demonstrated an association of resveratrol-induced changes in fasting glucose with improvements in endothelial vasodilator function, which warrants further investigation. The low dose of resveratrol was well tolerated over a period of 12 months without apparent side effects. Our findings support the adoption of resveratrol as a low-cost, effective intervention to help counteract the age and menopause-related accelerated cognitive decline in our ageing population. Subsequent publications from this study will report effects of resveratrol on bone health, physical function and quality of life measures which, collectively, will establish resveratrol as a viable intervention to promote healthy ageing in women. 

## Figures and Tables

**Table 1:** Cognitive domains and component tasks in the neuropsychological test battery.

Cognitive Domains	NIH-ToolBox Assessment	Other Assessment
Processing speed	Pattern Comparison Speed Test	Trail Making Task A
Language	Picture Vocabulary Test Oral Reading Recognition Test	
Working memory	List Sorting Working Memory Test	Forward Spatial Span test
Episodic memory	Picture Sequence Memory Test	
Verbal memory		Rey’s Auditory Verbal Learning Test (immediate recall and 30-minute delayed recall)
Cognitive flexibility	Dimensional Change Card Sort Test Flanker Inhibitory Control and Attention Test	Trail Making Task B

**Table 2:** Participant baseline characteristics (*n* = 146).

Participant’s Characteristics	Total (*n* = 146)	Placebo (*n* = 73)	Resveratrol (*n* = 73)
Age (years)	64 ± 1	64 ± 1	64 ± 1
Years since cessation of menses	15 ± 1	15 ± 1	15 ± 1
Years of education	17 ± 0.3	17 ± 0.5	17 ± 0.5
ACE-III score (%)	93 ± 0.5	93 ± 0.7	93 ± 0.5
BMI (kg/m^2^)	25.6 ± 0.3	25.8 ± 0.5	25.4 ± 0.5
Systolic blood pressure (mmHg)	124 ± 1	125 ± 2	123 ± 2
Diastolic blood pressure (mmHg)	68 ± 1	69 ± 1	67 ± 1
Large artery compliance (mL/mmHg×10)	12.6 ± 0.36	12.1 ± 0.38	13.1 ± 0.67
Small artery compliance (mL/mmHg×100)	3.7 ± 0.17	3.7 ± 0.29	3.5 ± 0.18

**Table 3:** Participants’ z-scores for each cognitive domain and individual cognitive tests between resveratrol and placebo groups following 12 months supplementation.

	Month 0		Month 12	
Cognitive Domains	Placebo (*n* = 73)	Resveratrol (*n* = 73)	Placebo (*n* = 66)	Resveratrol (*n* = 63)
● Component Tasks				
**Processing Speed**	0.05 ± 0.09	−0.08 ± 0.10	0.23 ± 0.10	0.24 ± 0.09 *
● PCT	0.08 ± 0.12	−0.13 ± 0.13	0.15 ± 0.13	0.32 ± 0.11 *
● TMT A	0.02 ± 0.13	−0.02 ± 0.11	0.31 ± 0.11	0.16 ± 0.11
**Language**	−0.03 ± 0.10	0.03 ± 0.11	0.04 ± 0.11	0.09 ± 0.11
● PVT	−0.09 ± 0.12	0.09 ± 0.12	0.05 ± 0.13	0.20 ± 0.13
● ORR	0.02 ± 0.12	−0.02 ± 0.12	0.04 ± 0.10	−0.02 ± 0.11
**Episodic Memory**	−0.49 ± 0.09	−0.47 ± 0.10	−0.27 ± 0.10	−0.19 ± 0.11
● PSM	−0.49 ± 0.09	−0.47 ± 0.10	−0.27 ± 0.10	−0.19 ± 0.11
**Verbal Memory**	0.001 ± 0.1	−0.001 ± 0.1	0.17 ± 0.12	0.12 ± 0.10
● RAVLT immediate	0.02 ± 0.07	−0.02 ± 0.07	0.07 ± 0.06	0.07 ± 0.06
● RAVLT delayed	−0.02 ± 0.15	0.02 ± 0.15	0.28 ± 0.19	0.17 ± 0.16
**Working Memory**	0.04 ± 0.10	−0.04 ± 0.10	0.06 ± 0.09	0.05 ± 0.09
● LSWM	0.11 ± 0.11	−0.11 ± 0.12	0.08 ± 0.14	−0.03 ± 0.13
● FSS	−0.04 ± 0.12	0.04 ± 0.11	0.06 ± 0.12	0.12 ± 0.10
**Cognitive Flexibility**	−0.03 ± 0.12	0.03 ± 0.08	0.78 ± 0.08	0.82 ± 0.07 *
● DCCS	−0.05 ± 0.14	0.05 ± 0.09	0.10 ± 0.13	0.23 ± 0.10
● FICA	−0.08 ± 0.12	0.08 ± 0.11	0.22 ± 0.11	0.26 ± 0.12
● TMT performance	0.02 ± 0.21	−0.02 ± 0.19	2.01 ± 0.10	1.97 ± 0.11
**Overall Cognitive Performance**	−0.08 ± 0.07	−0.09 ± 0.05	0.17 ± 0.06	0.18 ± 0.05 *

NOTE. * Significant treatment change between resveratrol and placebo, Analysis of Variance, *P* < 0.05. Abbreviations: DCCS = Dimensional Change Card Sort, PVT = Picture Vocabulary, FICA = Flanker Inhibitory and Control Attention, PSM = Picture Sequence Memory, PCT = Pattern Comparison Speed, LSWM = List Sorting Working Memory, ORR = oral reading recognition, RAVLT = Rey’s Auditory Verbal Learning Test, FSS = Forward Spatial Span, TMT = Trail Making Task.

**Table 4:** Indices of cerebrovascular function between resveratrol and placebo groups at baseline and after 12 months of supplementation. Data are presented as mean ± SEM.

Cerebral Hemodynamics	Month 0		Month 12	
**Resting conditions**	**Placebo (*n* = 51)**	**Resveratrol (*n* = 47)**	**Placebo (*n* = 51)**	**Resveratrol (*n* = 47)**
Systolic blood flow velocity (cm/s)	81.3 ± 2.7	76.0 ± 2.9	71.8 ± 1.9	76.2 ± 3.3
Diastolic blood flow velocity (cm/s)	36.0 ± 1.4	32.7 ± 1.3	30.9 ± 0.95	32.8 ± 1.4
Mean blood flow velocity (cm/s)	53.8 ± 1.9	49.6 ± 1.9	46.6 ± 1.3	49.9 ± 2.1
Pulsatility index	0.84 ± 0.02	0.87 ± 0.03	0.88 ± 0.02	0.86 ± 0.02
**Cerebrovascular responses to hypercapnia** (%)	**Placebo (*n* = 44)**	**Resveratrol (*n* = 41)**	**Placebo (*n* = 44)**	**Resveratrol (*n* = 41)**
	45.8 ± 1.7	46.4 ± 2.3	47.2 ± 2.03	48.9 ± 2.05
**Neurovascular coupling capacity (%)**	**Placebo (*n* = 45)**	**Resveratrol (*n* = 46)**	**Placebo (*n* = 45)**	**Resveratrol (*n* = 46)**
Processing speed	19.2 ± 1.4	19.6 ± 1.4	15.4 ± 1.3	16.9 ± 1.2
Language	14.0 ± 1.1	12.0 ± 0.97	11.9 ± 0.95	11.6 ± 0.63
Episodic memory	16.6 ± 1.3	14.5 ± 1.2	13.5 ± 1.07	14.2 ± 1.2
Verbal memory	15.9 ± 1.2	16.2 ± 1.02	12.6 ± 0.89	12.4 ± 0.85
Working memory	15.5 ± 1.02	14.2 ± 0.94	13.4 ± 0.83	13.0 ± 0.64
Cognitive flexibility	16.1 ± 0.94	14.3 ± 0.92	11.7 ± 0.77	13.5 ± 0.74
Overall cognition	16.4 ± 0.73	15.0 ± 0.63	12.8 ± 0.69	13.5 ± 0.59

**Table 5:** Cardiometabolic markers between resveratrol and placebo groups at baseline and after 12 months of supplementation. Data are presented as mean ± SEM.

	Month 0		Month 12		Δ Month 12–Month 0		
Fasting Serum Biomarkers	Placebo (*n* = 65)	Resveratrol (*n* = 59)	Placebo (*n* = 65)	Resveratrol (*n*= 59)	Placebo (*n* = 65)	Resveratrol (*n* = 59)	*P*-Value
Glucose (mmol/L)	5.0 ± 0.07	5.0 ± 0.07	5.0 ± 0.06	5.0 ± 0.06	0.05 ± 0.06	−0.00 ± 0.04	0.499
Insulin (mIU/L)	7.2 ± 0.46	7.9 ± 0.61	8.0 ± 0.42	8.1 ± 0.59	0.82 ± 0.42	0.16 ± 0.50	0.308
HOMA-IR	1.6 ± 0.12	1.8 ± 0.16	1.8 ± 0.11	1.8 ± 0.14	0.20 ± 010	0.01 ± 0.12	0.236
Triglycerides (mmol/L)	1.1 ± 0.06	1.2 ± 0.07	1.2 ± 0.06	1.3 ± 0.07	0.08 ± 0.04	0.10 ± 0.05	0.800
Total cholesterol (mmol/L)	5.7 ± 0.11	5.6 ± 0.18	5.7 ± 0.12	5.6 ± 0.18	−0.03 ± 0.07	−0.01 ± 0.08	0.901
LDL-cholesterol (mmol/L)	3.7 ± 0.11	3.5 ± 0.17	3.6 ± 0.11	3.5 ± 0.17	−0.08 ± 0.07	−0.03 ± 0.06	0.561
HDL-cholesterol (mmol/L)	1.6 ± 0.04	1.6 ± 0.05	1.6 ± 0.05	1.6 ± 0.05	0.02 ± 0.02	−0.03 ± 0.02	0.168
Hs-CRP (mg/L)	2.0 ± 0.24	2.4 ± 0.67	2.2 ± 0.43	2.5 ± 0.34	0.22 ± 0.43	0.10 ± 0.54	0.862

Abbreviations: HOMA-IR = Homeostatic Model Assessment of Insulin Resistance, LDL-cholesterol = low-density lipoprotein, HDL = high-density lipoprotein, Hs-CRP = high sensitivity C-reactive protein.

## Supplementary Materials

The following are available online at https://www.mdpi.com/2072-6643/12/3/828/s1, Table S1: Blood pressure (BP) and arterial compliance between resveratrol and placebo groups at baseline and after 12 months of supplementation. Data are presented as mean ± SEM.
