# Supplementary material for: Sustained Cerebrovascular and Cognitive Benefits of Resveratrol in Postmenopausal Women
Source: Nutrients. 2020 Mar 20;12(3):828. doi: 10.3390/nu12030828 (PMC7146200; doi:10.3390/nu12030828)
Supplement: Supplementary file 1 [file nutrients-12-00828-s001.pdf]

Supplementary Table 1: Blood pressure (BP) and arterial compliance between resveratrol and placebo groups at baseline and after 12 months of supplementation. Data are presented as mean ± SEM.

**Systemic vascular function**	**Month 0**		**Month 12**		**Δ Month 12 – Month 0**		***P*-value**
	**Placebo (*n* = 66)**	**Resveratrol (*n* = 61)**	**Placebo (*n* = 66)**	**Resveratrol (*n*= 61)**	**Placebo (*n* = 66)**	**Resveratrol (*n* = 61)**	
Systolic BP (mmHg)	125 ± 2	124 ± 2	123 ± 2	124 ± 2	-2.2 ± 1.5	-0.24 ± 1.4	0.329
Diastolic BP (mmHg)	69 ± 1	68 ± 1	68 ± 1	69 ± 1	0.02 ± 0.73	1.27 ± 0.64	0.219
Large artery compliance (ml/mmHg×10)	12.1 ± 0.38	13.1 ± 0.67	12.6 ± 0.76	11.7 ± 0.46	0.43 ± 0.71	-1.4 ± 0.63	0.053
Small artery compliance (ml/mmHg×100)	3.7 ± 0.29	3.5 ± 0.18	3.6±0.27	3.4±0.20	-0.10 ± 0.18	-0.03 ± 0.19	0.799
